# Monoclonal Antibodies in the Management of Familial Hypercholesterolemia: Focus on PCSK9 and ANGPTL3 Inhibitors

**DOI:** 10.1007/s11883-021-00972-x

**Published:** 2021-10-26

**Authors:** Angela Pirillo, Alberico L. Catapano, Giuseppe D. Norata

**Affiliations:** 1 Center for the Study of Atherosclerosis, E. Bassini Hospital, Cinisello Balsamo, Milan, Italy; 2 IRCCS MultiMedica, Sesto S. Giovanni, Milan, Italy; 3 Department of Pharmacological and Biomolecular Sciences, University of Milan, Milan, Italy

**Keywords:** Monoclonal antibodies, PCSK9, ANGPTL3, Familial hypercholesterolemia

## Abstract

**Purpose of Review:**

Familial hypercholesterolemia (FH) is a monogenic disorder characterized by high plasma levels of low-density lipoprotein cholesterol (LDL-C) since birth and a high risk of premature cardiovascular disease. The genetic defect is carried in only one allele in heterozygous FH (HeFH) or in both in the most severe homozygous FH (HoFH). Current guidelines recommend to reduce substantially LDL-C levels in these high-risk patients, with the need to use association therapy combining agents with different mechanisms of action. As most cases of FH are attributable to mutations in the gene encoding the low-density lipoprotein receptor (LDLR), statins, even in combination with ezetimibe, are less effective in reducing LDL-C plasma levels in FH patients, who require a more intensive approach with additional lipid-lowering agents. Additional targets playing key roles in regulating LDL-C levels are represented by PCSK9 and ANGPTL3.

**Recent Findings:**

Two monoclonal antibodies (mAbs) targeting PCSK9, evolocumab and alirocumab, significantly reduce LDL-C levels in HeFH patients. In patients with HoFH, the efficacy of mAbs to PCSK9 is strictly related to the presence of a residual LDLR activity; thus, patients carrying null mutations do not respond to the therapy with these mAbs, whereas some effects can be appreciated in HoFH bearing defective mutations. Conversely, evinacumab, the mAb targeting ANGPTL3, is highly effective in reducing LDL-C levels even in HoFH patients carrying null *LDLR* mutations, thanks to its LDLR-independent mechanism of action.

**Summary:**

Monoclonal antibodies inhibiting PCSK9 have shown a robust effect in FH patients presenting a residual LDLR activity, while ANGPTL3 inhibitors appear to be promising even in patients carrying null *LDLR* mutations.

## Introduction

Familial hypercholesterolemia (FH) is a monogenic disease characterized by elevated plasma levels of LDL-C since birth, resulting in an increased risk of premature cardiovascular disease. Most cases of FH are attributable to mutations in the *LDLR* gene (encoding the low-density lipoprotein receptor, LDLR) that account for more than 90% of cases, while a minor part has been linked to mutations in *APOB*, *PCSK9*, or in the *LDLRAP1* genes (in homozygosis). Mutations in *LDLR* gene range from defective (characterized by a residual receptor function) to null (with no functional receptor protein), and the severity of FH is also related to the presence of the genetic defect in only one allele (heterozygous FH, HeFH) or in both (homozygous FH, HoFH), with the latter having the lowest prevalence but the most severe phenotype. Current guidelines categorize individuals with FH as high or very high-risk patients (depending on the absence or presence of ASCVD or another major risk factor) and recommend to treat them to reduce their LDL-C levels at least by 50% and to reach an LDL-C goal below 55 mg/dl if at very high risk or below 70 mg/dl if at high risk [1]. Accordingly, the first approach always consists in a statin therapy, which can be combined with ezetimibe if the goal is not reached, and further also with a PCSK9 inhibitor.

The efficacy of statins in the management of hypercholesterolemia in FH is strictly related to the type of genetic defect underlying the clinical phenotype. In fact, as these drugs work by upregulating the expression of hepatic LDLR, in HeFH statins induce the transcription of both the mutated and the wild-type allele, thus resulting in an increased expression of functional LDLR in the liver (although at a lesser extent than in non-FH individuals) and the reduction of plasma LDL-C. In HoFH, the picture is more complicated due to the presence of mutations in both alleles. If the patient bears a defective mutation, implying the presence of a residual functionality of the mutated protein, statin therapy upregulates the expression of this poorly functional protein which, however, still contributes to reduce, at least in part, LDL-C levels. However, in the presence of null mutations, leading to the absence of any residual functional receptor, the effect of statins is blunted. This implies that these patients are poorly responders to statins and potentially to any other cholesterol-lowering drug requiring at least a residual LDLR function.

From a clinical perspective, the reduction in LDL-C plasma levels achieved in these individuals with statins, even in combination with ezetimibe, is however not enough to substantially reduce their CV risk, thus casting for a more intensive approach with lipid-lowering agents such as monoclonal antibodies improving LDL-C catabolism through either LDLR-dependent or independent mechanisms.

The aim of this review is to discuss current options and clinical efficacy of monoclonal antibodies targeting PCSK9 or ANGPTL3 in FH patients according to the genetic mutations underlying the disease.

## Monoclonal Antibodies Targeting PCSK9

PCSK9 is a serine protease playing a crucial role in LDLR recycling and therefore modulates circulating plasma LDL-C levels.

PCSK9 is mainly secreted from the liver and binds extracellularly to LDLR. Following internalization, the presence of PCSK9 bound to LDLR targets it to lysosomal degradation, thus preventing LDLR recycling at the cell surface. As a consequence, cells reduce their expression of LDLR and the uptake of LDL particles, with a consequent increase of LDL-C plasma levels [2]. The relevance of PCSK9 in atherosclerosis and related CVD has been established by the observation that subjects carrying loss-of-function mutation in *PCSK9* gene exhibit lower LDL-C levels and a substantial reduction in cardiovascular risk [3], while gain-of-function variants are associated with familial hypercholesterolemia and, as a consequence of the lifelong exposure to elevated levels of LDL-C, increased CV risk [4]. Accordingly, Mendelian randomization studies indicated that variants in *PCSK9* and *HMGCR* genes are associated with similar reductions in the risk of CVD per unit decrease in LDL-C levels [5]. These findings paved the road for the rapid development and approval of two monoclonal antibodies targeting PCSK9, evolocumab and alirocumab, recently followed by the approval of a PCSK9 siRNA [2, 6].

Evolocumab and alirocumab have undergone extensive clinical development programs showing definitely their high efficacy in reducing LDL-C levels and the risk of CVD along with their safety [7]. Evolocumab has been evaluated in the PROFICIO (Program to Reduce LDL-C and Cardiovascular Outcomes Following Inhibition of PCSK9 in Different Populations) clinical trial program in a broad variety of patient populations, either as monotherapy or as add-on to ongoing lipid-lowering therapy, with the FOURIER trials showing the benefit of using evolocumab on cardiovascular events [8]. Efficacy and safety of alirocumab were tested in the ODYSSEY clinical trial program in different groups of patients, and the positive clinical impact was proven by the ODYSSEY OUTCOMES trial in patients with acute coronary syndrome [9]. Both outcome trials reported a 15% relative risk reduction in the primary endpoint.

Evolocumab and alirocumab were extensively and successfully tested in patients with FH; as expected on the basis of the mechanism of action, the efficacy in FH was dependent on the allele status and the severity of mutation underlying FH [10].

### PCSK9i in HeFH

**Fig. 1:**
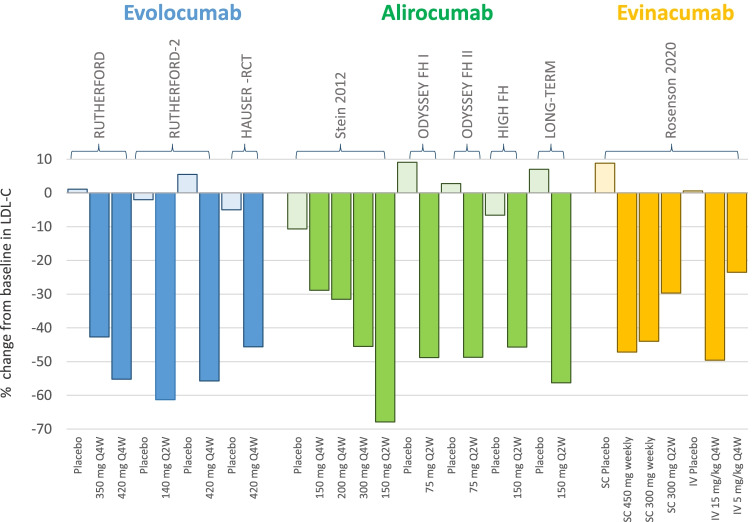
Mean percent changes from baseline in LDL-C levels in HeFH patients treated with evolocumab, alirocumab, or evinacumab (or placebo). Changes were evaluated at week 12 (RUTHERFORD, RUTHERFORD-2, HAUSER-RCT, Stein 2012, ODYSSEY FH I and FH II, ODYSSEY HIGH FH), week 24 (ODYSSEY LONG-TERM), or week 16 (Rosenson 2020)

The two RUTHERFORD trials [11, 12] have assessed the effect of evolocumab in patients with HeFH (Fig. 1). In the phase 2 RUTHERFORD trial, HeFH patients received evolocumab 350 mg or 420 mg, or placebo Q4W for 12 weeks. Significant reductions in LDL-C levels were observed with both evolocumab doses (− 42.7% with 350 mg and − 55.2% with 420 mg compared with a 1.1% increase in the placebo arm) [11]. Most patients treated with evolocumab (95%) achieved reductions in LDL-C of at least 15%, and 52% of them had their levels reduced by ≥ 50% [11]. In the phase 3 RUTHERFORD-2 trial, 331 HeFH patients were assigned to evolocumab 140 mg Q2W, evolocumab 420 mg Q4W, or placebo for 12 weeks [12]. Both evolocumab doses were highly effective in reducing LDL-C compared with placebo (treatment differences: 59.2% and 61.3%, with 140 mg Q2W and 420 mg Q4W, respectively). The analysis of changes in LDL-C levels based on causative mutations showed that, compared with placebo, patients carrying an LDLR null mutation showed reductions of 61.1% and 55.1% with 140 mg Q2W and 420 mg Q4W, respectively, that were not different from those observed in patients carrying an LDLR defective mutation (49% and 66%, respectively), or with unclassified LDLR status (62% and 63%) [12] (Fig. 1). In 13 patients carrying the same mutation on LDLR evolocumab reduced LDL-C levels from 27% to 83% [12]; the genetic analysis, furthermore, identified 7 patients being HoFH or compound HeFH in whom 68% (range 40%–82%) and 48% (range 38%–64%) reductions in LDL-C levels were observed with 140 mg Q2W and 420 mg Q4W, respectively [12]. The HAUSER-RCT trial assessed the efficacy of 420 mg Q4W evolocumab in 157 pediatric (10–17 years) patients with HeFH, with LDL-C levels being reduced by 44.5% compared with a 6.2% reduction observed in the placebo arm at week 24 (treatment difference − 38.3%) [13••].

A phase 2 study with alirocumab at different doses and regimens added to statins with or without ezetimibe in HeFH patients showed significant reductions in LDL-C levels, ranging from 28.9% to 67.9% (compared with a 10.6% reduction with placebo) at week 12 [14]. In the ODYSSEY FH I and II phase 3 trials, HeFH patients (486 in FH I and 249 in FH II) with inadequately controlled LDL-C levels despite maximally tolerated dose of statin with or without other lipid-lowering drugs were randomized to receive placebo or alirocumab 75 mg Q2W (increased to 150 Q2W if LDL-C persisted at ≥ 70 mg/dl after 12 weeks) (Fig. 1) [15]. At week 24, LDL-C was reduced by 57.9% in FH I and 51.4% in FH II, and the reductions were maintained through week 78 [15]. A large part of patients achieved LDL-C < 70 mg/dl at week 24 (59.8% in FH I and 68.2% in FH II) [15]. Similar results were reported in the ODYSSEY HIGH FH trial, in which HeFH patients having baseline LDL-C levels ≥ 160 mg/dl showed a significant reduction when treated with alirocumab 150 Q2W compared with placebo (− 39.1%) at week 24, that was maintained through week 78 [16], and in the ODYSSEY LONG TERM trial, showing that HeFH patients responded to alirocumab 150 mg Q2W similarly to non-HeFH patients (difference vs placebo: − 63.2% and − 61.5%, respectively) [17], an efficacy that was confirmed by the open-label extension trial up to 4 years (Fig. 1) [18]. The analysis of alirocumab efficacy according to the underlying genetic defect showed that patients responded substantially in a similar way to alirocumab treatment independently of the mutation, with LDL-C reductions from 48.3% to 60.7%, in patients heterozygous either for LDLR-defective or negative mutations, or carrying APOB-defective mutations [19••] (Fig. 1). In the ODYSSEY KIDS trial, alirocumab was tested also in 4 pediatric HeFH cohorts (8–17 years), at different doses according to body weight [20]; after 8 weeks of treatment, reductions in LDL-C up to 46% were observed.

### PCSK9 in HoFH

**Fig. 2:**
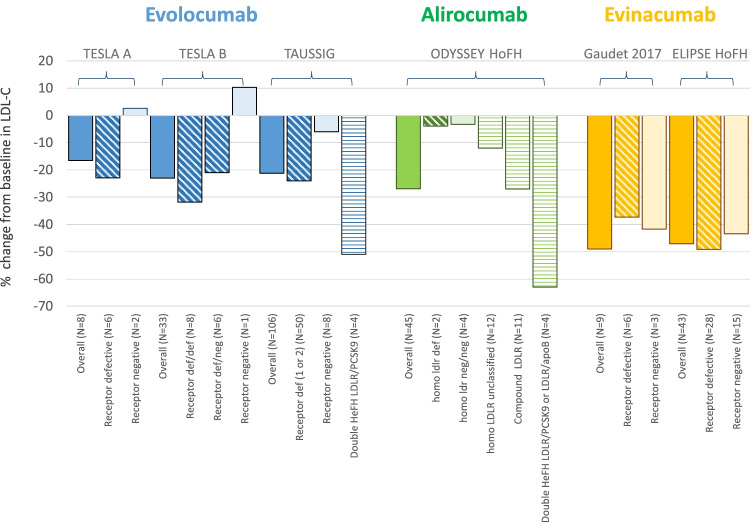
Mean percent changes from baseline in LDL-C levels in HoFH patients treated with evolocumab, alirocumab, or evinacumab, by mutation status. Changes were evaluated at week 12 (TESLA A, TESLA B, TAAUSSIG, ODYSSEY HoFH), week 4 (Gaudet 2017), or week 24 (ELIPSE HoFH)

As monoclonal antibodies targeting PCSK9 are anticipated to act by increasing hepatic LDLR expression, it is expected that HoFH patients may present a reduced response, particularly those carrying LDLR null mutations. To answer this question, several studies have evaluated the lipid-lowering activity of evolocumab and alirocumab in patients with HoFH and assessed analyses based on the causative mutation (Fig. 2). The TESLA part A trial was an open-label, single arm study that recruited 8 LDLR negative or defective HoFH patients treated with evolocumab 420 mg Q4W for 12 weeks and then 420 mg Q2W for an additional 12 weeks; reductions in LDL-C by 13.3% with the Q4W dosing and by 16.9% with the Q2W dosing were reported; the two patients with LDLR-negative activity did not show any reduction in LDL-C [21] and a wide range of variability was observed in defective patients. The TESLA part B trial, in which 49 patients were treated with evolocumab 420 mg or placebo Q4W for 4 weeks on top of the ongoing LLT, reported a 30.9% reduction in LDL-C compared to baseline [22]. When analyzed according to LDLR mutation status, patients with a receptor defective mutation in one or both alleles had a significant reduction in LDL-C levels (40.8% compared with placebo), whereas those carrying one defective and one negative mutation had a lower, although still significant, LDL-C reduction (− 24.5% compared with placebo) [22]. The patient with LDLR null mutations in both alleles and the patient with autosomal recessive HoFH did not respond to evolocumab treatment [22]. Among 8 patients carrying the same LDLR mutation, a high variability in the response to evolocumab was observed, with reductions ranging from 7.1 up to 56.0% [22].

These findings have been confirmed by the TAUSSIG trial (Fig. 2) [23]. In this study, 300 patients with HoFH (106) or severe HeFH were treated with evolocumab 420 mg Q4W or Q2W if on lipoprotein apheresis for a median of 4.1 years; LDL-C levels were reduced by 21.1% in patients with HoFH and by 54.9% in patients with severe HeFH, reductions that were maintained over the course of the study [23]. Among HoFH, in 48 patients evolocumab was uptitrated from 420 mg Q4W to 420 mg Q2W, resulting in a higher reduction in LDL-C levels (pre-titration: − 19.6%, post-titration − 29.7%) [23]. An analysis based on the underlying mutation showed a high variability in the response to evolocumab [24]. A recent analysis showed that, among patients having identical mutations in *LDLR*, cell surface LDLR expression is highly variable, which likely drives their individual response to evolocumab [25••]. Of note, even subjects carrying the same mutation on the LDLR gene exhibit a certain degree of variability in plasma lipid and lipoproteins that can be attributed to additional genetic factors (such as polymorphisms in other genes that are known to affect plasma lipids), environmental factors, and their interaction. Such phenotypic heterogeneity is believed to be responsible, at least in part, for the observed variability in the response to lipid-lowering treatment.

A wide range of LDL-C reduction, (21.7% to 63.9%) has (ranging from 21.7% to 63.9%) been observed following the treatment with alirocumab 75/150 mg or 150 mg Q2W in patients with double heterozygous, compound heterozygous, or homozygous FH [26]. The treatment of HoFH with alirocumab 150 mg Q2W in the ODYSSEY HoFH trial resulted in a substantial reduction in LDL-C levels at week 12 (difference vs placebo: − 35.6%) (Fig. 2) [27••]. At the end of the double-blind phase, all patients entered a 12-week open-label treatment period and received 150 mg Q2W: the mean reduction in LDL-C from baseline to week 24 with alirocumab was 27.3% (67.9 mg/dl), with patients treated with alirocumab during the double-blind phase showing greater LDL-C reductions than those who received placebo (30.7% vs 20.6%) [27••]. LDL-C reductions were highly variable, but mostly low or absent in patients carrying null/null LDLR mutations.

## Monoclonal Antibody Targeting ANGPTL3

Angiopoietin-like 3 (ANGPTL3) is an endogenous inhibitor of lipoprotein lipase (LPL) and endothelial lipase (EL), two enzymes playing an important role in lipoprotein metabolism [28]. Individuals carrying loss-of-function mutations in the *ANGPTL3* gene present hypolipidemia with reduced levels of triglycerides (TG) and LDL-C [29, 30••, 31]. Subjects with low ANGPTL3 levels in the population present a significantly reduced risk of myocardial infarction compared to subjects with elevated ANGPTL3 plasma levels [29, 30••]. These findings suggest that ANGPTL3 could represent a potential pharmacological target for the treatment of dyslipidemic patients. Most importantly, the initial findings with ANGPTL3 inhibitors demonstrated that cholesterol-lowering effects of ANGPTL3 are independent of LDLR modulation, paving the way for testing the inhibition of ANGPTL3 in HoFH patients, and particularly in those carrying null *LDLR* mutations [32]. Different strategies are under clinical evaluation to inhibit ANGPTL3; although most of the data so far are available for the monoclonal antibody evinacumab, a gene silencing approach with vupanorsen is also under evaluation [33].

In healthy volunteers, evinacumab reduced LDL-C (up to 23%) and TG (up to 76%) levels [30••]. Evinacumab has been tested at different doses and regimens in 272 patients with refractory hypercholesterolemia (including 116 with HeFH) (Fig. 1). At week 16, LDL-C levels were reduced substantially in all groups receiving evinacumab compared with placebo (56.0% with subcutaneous evinacumab 450 mg weekly, 52.9% with subcutaneous evinacumab 300 mg weekly, 38.5% with subcutaneous evinacumab 300 mg Q2W, 50.5% with intravenous evinacumab 15 mg/kg Q4W, and 24.2% with intravenous evinacumab 5 mg/kg Q4W) [34••].

A single group, open-label study involving nine HoFH patients showed that evinacumab added to background lipid-lowering therapy further reduced LDL-C level by 49%; due to the heterogeneity of genetic defects underlying this condition, there was a broad variability in LDL-C level reductions that ranged from 25% to 90% at week 4 [35]. Interestingly, three patients with null/null mutations (2 homozygotes and 1 compound heterozygote) had significant, although different, responses to evinacumab (26%, 42%, and 44%, respectively) (Fig. 2) [35]. The phase 3 ELIPSE HoFH trial confirmed the efficacy of evinacumab in HoFH patients already under intensive treatment with available therapies: LDL-C levels were overall reduced by 47.1% from baseline (compared with a 1.9% increase with placebo) after 24 weeks in 65 HoFH patients; evinacumab reduced LDL-C levels both in patients with null/null variants and in those with non-null variants (Fig. 2) [36••]. A recent study evaluated the effect of evinacumab treatment also in two young HoFH patients (aged 12 and 16) carrying null/null LDLR variants [37••]. Both patients were on apheresis and treated with statin + ezetimibe; the addition of evinacumab to the current therapy substantially lowered LDL-C levels pre-apheresis (55% and 56.6%) and post-apheresis (45% and 43%) [37••]. Notably, both patients experienced a profound, virtually complete plaque regression upon highly intensive LDL-C lowering [37••], a change which was never appreciated in adult HoFH patients following the treatment with intensive lipid-lowering therapy [38]. This finding suggests that an early intervention with a therapy that reduces significantly LDL-C levels not only can impact coronary plaque formation, but can also revert atherosclerosis, an effect that can be largely attributed to the characteristics of atherosclerotic plaque at its young age.

These results, beyond demonstrating a robust effect in reducing LDL-C in patients who poorly benefit from classical lipid-lowering therapies as well as from PCSK9 inhibitors, confirm the LDLR-independent efficacy of evinacumab and call for a better understanding of the molecular mechanism responsible for this effect.

At the molecular level, carriers of ANGPTL3 loss-of-function mutations exhibit a decreased production rate of VLDL apoB [39], suggesting the possibility that the reduction in LDL-C levels observed in HoFH patients treated with evinacumab could be the consequence of a reduced production of lipoproteins. A recent small study in 4 HoFH patients [40] examined apoB-containing lipoprotein kinetic parameters before and after treatment with evinacumab and observed that ANGPTL3 inhibition was associated with an increase in the fractional catabolic rate of IDL apoB and LDL apoB, suggesting that evinacumab lowers LDL-C predominantly by increasing apoB-containing lipoprotein clearance from the circulation.

How LPL and/or EL re-activation following ANGPTL3 inhibition may increase apoB-containing lipoprotein clearance from the circulation in an LDLR-independent manner is still unknown. The observation that evinacumab de-repress EL, resulting in an extensive remodelling of VLDL, which accelerates its clearance from the circulation and reduces LDL-C levels [41], particularly in the absence of a functional LDLR, suggests the hypothesis that EL-modified VLDL may be cleared through multiple redundant receptors [41].

## Conclusions

Until now, the management of severe cases of FH, including HoFH and HeFH with null mutations, has been limited by the mechanism of action of most lipid-lowering therapies, which act by increasing the expression of LDLR. These patients poorly benefit from these therapies; apheresis is the most effective approach, although with a relevant impact in life quality, while lomitapide, an MTP inhibitor, should be handled with care to limit lipid accumulation in the liver. In this scenario, monoclonal antibodies inhibiting PCSK9 have shown a robust effect in FH patients presenting a residual LDLR activity, while ANGPTL3 inhibitors appear to be promising in patients carrying null LDLR mutations. Of note, lipid-lowering therapies based on gene silencing of hepatic PCSK9 or ANGPTL3 are under development [42]. Of note, inclisiran, a siRNA that inhibits the hepatic synthesis of PCSK9 [2, 6], was recently shown to reduce LDL-C levels in adults with HeFH similarly to PCSK9 mAbs, but with a less frequent dosing regimen (twice a year administration), which has the potential to enhance the patient adherence to the treatment [43]. As circulating levels of PCSK9 and ANGPTL3 are largely contributed by the liver, it is expected that both mAb and gene silencing approaches would act by controlling the two targets at this level. It is known that PCSK9 can also be produced locally in the endocrine pancreas or the heart [44, 45]; the production at this level, however, should not be influenced by either mAbs or gene silencing. Of note, the pro-atherosclerotic effect of PCSK9 is not exclusively due to its role in modulating LDL-C plasma levels, as this protein can locally increase vascular inflammation and contribute to atherosclerotic plaque progression independently of circulating lipid changes [46]. Whether different pharmacological approaches to inhibit PCSK9 (mAb *vs* gene silencing) may differently impact the intraplaque effects of PCSK9 remains to be determined.

Thus, there are still some gaps related to the understanding of PCSK9 biology beyond the liver and ANGPTL3 biology beyond LDLR that need to be addressed to safely improve the management of HoFH patients with monoclonal antibodies targeting PCSK9 or ANGPTL3.
